# Increase in Serotype 6C Pneumococcal Carriage, United Kingdom

**DOI:** 10.3201/eid1601.090650

**Published:** 2010-01

**Authors:** Anna S. Tocheva, Johanna M.C. Jefferies, Myron Christodoulides, Saul N. Faust, Stuart C. Clarke

**Affiliations:** University of Southampton School of Medicine, Southampton, UK (A.S. Tocheva, J.M.C. Jefferies, M. Christodoulides, S.C. Clarke); University of Southampton Wellcome Trust Clinical Research Facility, Southampton (S.N. Faust)

**Keywords:** Heptavalent pneumococcal conjugate vaccine, multilocus sequence typing, pneumococcal typing, bacteria, respiratory infections, United Kingdom, letter

**To the Editor:** *Streptococcus pneumoniae* is a major human pathogen. In 2007, Park et al. identified a novel serotype, 6C (*1*), which emerged from serotype 6A. A study of children in the Netherlands who had not previously received a pneumococcal vaccine found low prevalence of this newly identified serotype before the heptavalent pneumococcal conjugate vaccine Prevnar/Prevenar (PCV7) (Wyeth, Taplow, UK) was introduced (*2*). Studies have shown cross-protection between vaccine serotype 6B and vaccine-related serotype 6A. However, PCV7 elicits no cross-protection against serotype 6C.

The potential exists for the emergence of nonvaccine serotypes or novel clones. These serotypes and clones may be better adapted to colonize the nasopharynx, evade the human immune response, and cause disease. A recent study showed an increase in prevalence of serotype 6C pneumococci in children and a corresponding decrease in serotype 6A after introduction of PCV7 (*3*). We studied the underlying genetic basis for expansion of serotype 6C. Initial data from an ongoing study of pneumococcal carriage are presented.

This study was reviewed and approved by the Southampton and South West Hampshire Research Ethics Committee (B) (reference 06/Q1704/105). A total of 697 nasopharyngeal swab specimens were collected from unselected (not selected by a method) children <4 years of age in the pediatric outpatient department of a large teaching hospital in the United Kingdom. Samples were obtained during October 2006–March 2007, during implementation of PCV7 in the infant immunization schedule of the United Kingdom. During October 2007–March 2008, a total of 202 pneumococci were isolated. All pneumococci were characterized by serotype and genotype.

In the first year of this study, we identified 3 (3.1%) serotype 6C pneumococci belonging to 3 sequence types (STs): ST65, ST1714, and ST1692 (Appendix Figure). ST1714 and ST 1692 shared a common clonal complex. Only ST 65 was shared between serotype 6C and serotype 6A. In the second year, we identified 14 (13.6%) serotype 6C pneumococci belonging to 6 STs (Appendix Figure). Two of these STs, of the same ST, were from siblings. Three of them (ST1692 [n = 8], ST1714 [n = 2], and ST395 [n = 1]) were members of a common clonal complex with a predicted founder of ST395. Each of the remaining 3 STs (ST398, ST1862, and ST3460) was isolated only once. One serotype 6A isolate of ST1692 was also observed.

No serotype 6C ST65 was observed in the second year. We isolated more serotype 6C pneumococci in year 2 than in year 1 (p<0.01), which was explained mostly by a large increase in ST1692 (p<0.03) (Appendix Figure). A recent study by Nunes et al. reported serotype 6C ST1692 within a clonal complex that also included ST395 and ST1714 (*4*), and we identified the same clonal complex in year 2 of our study.

Our study showed a large increase in ST1692 in serotype 6C pneumococci during the implementation of PCV7 and an increase in serotype 6C. Depending on the extent of cross-protection between vaccine-related serotypes, introduction of conjugate vaccines could induce clearance or emergence of vaccine-related serotypes. This introduction could also contribute to their substitution with novel or existing serotypes that are better adapted to the ecologic niche. However, our data may only be relevant to carried pneumococci and not reflected in pneumococcal disease epidemiology. Nevertheless, the increase in serotype 6C pneumococci in the United Kingdom, which is supported by a similar observation in the United States (*3*), highlights the potential for emergence of serotypes not included in the current study and newly developed pneumococcal conjugate vaccines.

## Supplementary Material

Appendix Figure Genotypes of serotype 6C pneumococci isolated from children in 2006-2007 (year 1) and 2007-2008 (year 2), United Kingdom. ST, sequence type.
