# Supplementary figures and images for: Increase in Serotype 6C Pneumococcal Carriage, United Kingdom
Source: Emerg Infect Dis. 2010 Jan;16(1):154–5. doi: 10.3201/eid1601.090650 (PMC2874357; doi:10.3201/eid1601.090650)

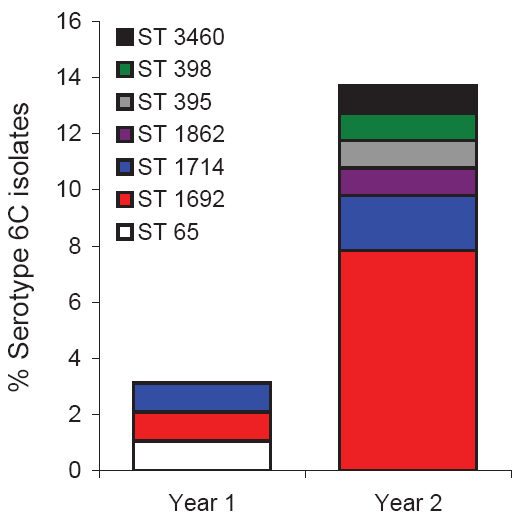

Supplement: Appendix Figure — Genotypes of serotype 6C pneumococci isolated from children in 2006-2007 (year 1) and 2007-2008 (year 2), United Kingdom. ST, sequence type. [file 09-0650_appF-s1.gif]
